# Editorial: Long-term effects of early-life manipulations: risks and advantages for neurodevelopment

**DOI:** 10.3389/fncel.2023.1193912

**Published:** 2023-04-20

**Authors:** Valentina Gigliucci, Matteo Di Segni, Rossella Ventura, Marco Battaglia

**Affiliations:** ^1^Division of Neuroscience, San Raffaele Hospital (IRCCS), Milan, Italy; ^2^Institute of Neuroscience, National Council of Research, Milan, Italy; ^3^Santa Lucia Foundation (IRCCS), Rome, Italy; ^4^Department of Psychology, Sapienza University of Rome, Rome, Italy; ^5^IRCCS San Raffaele, Rome, Italy; ^6^Department of Psychiatry, University of Toronto, Toronto, ON, Canada

**Keywords:** neurodevelopment, early-life events, long-term effects, critical windows, mental disorder (disease)

Both clinical and preclinical data show how crucial early life is to understand the neurobiological basis of many developmental processes and psychiatric conditions. Experimental manipulations that take place during this period of intense growth are often associated with long-lasting structural and functional brain responses to future environmental challenges, as well as distinct responses to pharmacological probing. While preclinical paradigms can only *model* real-life circumstances and conditions, they offer unparalleled control over the environment, and are immune to the intricacies of gene-environment correlations typical of human life (Battaglia et al., 2007; Luchetti et al., 2015). Also, early-life manipulations not only affect the mean response to a laboratory challenge: variance is often also equally altered. This indicates how individual differences matter for neurodevelopmentally-programmed responses, and the degree of departure from trajectories that one may consider strictly determined and invariant. Consequently, gathering deeper knowledge on how genetic and environmental components—alone and interactively—affect susceptibility/resilience to complex phenotypes is a decisive endeavor.

Finally, clinical trials of early-life pharmacological interventions for selected neurodevelopmental pathologies have been recently approved. This raises hopes that more conditions can be treated at infancy in a foreseeable future, with beneficial effects lasting several years, or possibly for the rest of a patient's lifetime.

By covering several, complementary aspects that stem from the issues outlined above, this Research Topic Issue provides several new pieces of knowledge on neurodevelopment. The papers address questions relevant to: gene-by-environment interaction, systemic changes in brain circuitry, longitudinal resilience/susceptibility toward adult psychopathology, and early systems/circuits manipulation to rescue behavioral functions and molecular fingerprints longitudinally.

Two contributions, one from Pisa et al. and the other from Cui et al., analyse the long-term repercussions of early deficiency vs. dispensation of certain compounds during development. Pisa et al. show how reduced oligosaccharides (HMOs, key chemicals that naturally occur in breast milk) availability affects cognitive development. By combining a genetic model (i.e. dams knocked-out for HMOs-synthesizing enzymes) with a cross-fostering procedure, the authors show the longitudinal association between reduced HMOs availability in childhood and impaired memory and attention at adulthood, possibly mediated by the serotoninergic system. Cui et al. show how a routine lab procedure such as anesthetization can longitudinally affect subjects with neurodevelopmental disorders, by evaluating the behavioral and molecular effects of multiple anesthetic (sevoflurane) exposures among BTBR mice, a model organism for autism spectrum disorders. Quite unexpectedly, not only did this treatment induce beneficial molecular outcomes on the Excitatory:Inhibitory (E/I) imbalance of these mice, by elevating the reduced inhibitory currents in pyramidal neurons, but also implemented mitochondrial respiration, and increased the BDNF/TrkB pathway components. Aside of the molecular changes, early sevoflurane treatment also ameliorated autistic-like traits with improvements in sociability and reduction of repetitive behaviors.

Since rearing conditions are a known critical environmental factor in shaping future responses to challenging stimuli, four contributions tackled the long-term impact of early-life events and experiences, such as stress or social neglect. The mini-review by Packard and Opendak provides a targeted overview of animal models of early adverse/insufficient caregiving. Within their attachment theory framework of reference, they focus on the role of social context in stress exposure. By highlighting dopaminergic circuitries and brain regions particularly susceptible to early adversities, they make a case for habenular dysfunction and its involvement in susceptibility to Major Depression and Schizophrenia. With their study in *Fmr1* knock-out mice, a model organism for Fragile X syndrome and Autism Spectrum Disorder, Petroni et al. investigate whether prenatal stress exerts genotype-dependent, long-term, age-specific behavioral effects. Their work shows that prenatal stress elicits some responses only in knock-out mice, implying that both genetic and environmental factors shape the long-lasting effects of prenatal stress. Demaili et al. investigate within a “two-(or multiple) hits” framework the age-dependent effects of stress exposure on behaviors mediated by the endocannabinoid system (CB1R; FAAH). By differentially exposing animals to early-life, or both early-life and adolescence stress, they tackle epigenetic DNA modifications (methylation) as a molecular mechanism by which experiences affect the transcriptional responses to new stressors faced later in life.

Moving onto cellular and molecular mechanisms, the mini-review by Pangrazzi and co-workers illustrates the pathways affected by enriched or impoverished rearing conditions across critical developmental windows, and the relevance for neuropsychiatric disorders. They propose a new, context-based behavioral adaptation hypothesis, whereby early-life experiences impinge on physiological mediators (e.g., neurotrophins, growth factors) to promote adaptation during critical time windows. Imbalances between resilience and susceptibility to stress can then arise, laying the foundation for a variety of psychiatric conditions.

Lastly, the contribution by Consorti et al. has high translational potential: they propose a non-invasive protocol to force long-term visual plasticity as a therapeutic strategy for amblyopia in adults. This is new to the field, as conventional occlusion therapies do not work well outside the critical window of juvenile visual cortex plasticity. The research team developed a protocol of active training based on visual perception learning (vPL) under binocular sight conditions in a rat model of amblyopia, and demonstrate its efficacy in favoring adult visual cortex plasticity and in eliciting stable, long-term recovery of visual functions.

A common thread is recognizable across these contributions: external influences and experiences have their greatest chance of shaping developmental trajectories during early-life, when neurodevelopment is at its highest sensitivity, and responses to environmental stimuli can be primed, often for the rest of an individual's life. In a larger contextualization, these studies suggest that to improve the population's future health, and to reduce the burden of psychiatric conditions on National Health Systems in the future, States should focus and invest massively so that children are raised out of poverty, with proper food provisions, and in enriched and stimulating environments. This acquires great relevance in today's society, where even in Western Countries an increasing number of families are finding themselves living just above or even below the poverty line, due to the constantly increasing cost of living.

## Author contributions

All authors listed have made a substantial, direct, and intellectual contribution to the work and approved it for publication.
